# Discordância entre Diretrizes Internacionais sobre Critérios de Prevenção Primária de Morte Súbita na Cardiomiopatia Hipertrófica

**DOI:** 10.36660/abc.20190161

**Published:** 2020-08-19

**Authors:** Beatriz Piva e Mattos, Fernando Luís Scolari, Henrique Iahnke Garbin

**Affiliations:** 1 Hospital de Clínicas de Porto Alegre Porto Alegre RS Brasil Hospital de Clínicas de Porto Alegre , Porto Alegre , RS - Brasil; 2 Universidade Federal do Rio Grande do Sul Faculdade de Medicina Porto Alegre RS Brasil Universidade Federal do Rio Grande do Sul - Faculdade de Medicina , Porto Alegre , RS - Brasil

**Keywords:** Cardiomiopatia Hipertrófica/genética, Hereditariedade, Morte Súbita Cardíaca, Arritmias Cardíacas, Síncope, Desfibriladores Implantáveis, Choque, Estudos de Coortes

## Abstract

**Fundamento:**

A estratificação de risco para morte súbita (MS) na cardiomiopatia hipertrófica (CMH) baseia-se em algoritmos distintos propostos pela diretriz norte-americana, *ACCF/AHA* 2011 e europeia, *ESC* 2014.

**Objetivo:**

Analisar o modelo *ESC* 2014 na determinação do risco de MS e indicação de cardiodesfibrilador implantável (CDI) em prevenção primária na CMH por meio de confrontação com a normativa norte-americana.

**Métodos:**

Foi avaliada uma coorte de pacientes com CMH, calculado o escore *ESC HCM-Risk-SCD* e analisada a concordância dos critérios de indicação de CDI entre as duas diretrizes pelo coeficiente de Kappa. O nível de significância adotado nas análises estatísticas foi de 5%.

**Resultados:**

Em 90 pacientes consecutivos, seguidos por 6±3 anos, o escore calculado foi de 3,2±2,5%. Os preditores que mais contribuíram para o cálculo nas faixas de baixo (1,88% [1,42-2,67]), médio (5,17% [4,89-5,70]) e alto risco (7,82% [7,06-9,19]) foram espessura parietal máxima do ventrículo esquerdo (1,60% [1,25-2,02] *;* 3,20% [3,18-3,36] *;* 4,46% [4,07-5,09]), diâmetro do átrio esquerdo (0,97% [0,83-1,21]; 1,86% [1,67-2,40]; 2,48% [2,21-3,51]) e idade (-0,91% [0,8-1,13]; -1,90% [1,12-2,03]; -2,34% [1,49-2,73]). O modelo europeu reduziu as recomendações de CDI em 32 (36%) pacientes. Entre os 43 (48%) em classe IIa pela *ACCF/AHA* , 8 (18%) migraram para IIb e 24 (56%) para III. Baixa concordância foi identificada entre as duas sistematizações, Kappa = 0,355, p = 0,0001. Dos 8 (9%) pacientes com MS ou choque apropriado, 4 (50%) atingiram indicação IIa pela *ACCF/AHA* , mas nenhum pela *ESC* .

**Conclusão:**

Baixa concordância foi identificada entre as diretrizes analisadas. O novo modelo reduziu as indicações de CDI, notadamente em classe IIa, mas deixou desprotegida a totalidade de pacientes com MS ou choque apropriado. (Arq Bras Cardiol. 2020; 115(2):197-204)

## Introdução

A cardiomiopatia hipertrófica (CMH) representa a doença cardiovascular de origem genética mais prevalente, com acometimento de um em cada 200 indivíduos. ^1^ A morte súbita (MS), atualmente estimada em 0,5 a 1% ao ano, incide em qualquer faixa etária, embora predomine entre jovens e atletas. ^2 - 4^

A estratificação de risco para MS na CMH constitui a base para indicação de cardiodesfibrilador implantável (CDI), única modalidade considerada capaz de modificar o prognóstico da doença. ^4 - 7^ Em pacientes com parada cardiorrespiratória prévia a indicação é consensual; contudo, em relação à prevenção primária, permanecem interrogações. Cinco fatores individualizados em estudos longitudinais e validados em metanálise são reconhecidos como preditores independentes de MS: história familiar, síncope inexplicada, espessura parietal máxima do ventrículo esquerdo (EPMVE) ≥ 30 mm, taquicardia ventricular não sustentada (TVNS) e resposta anormal da pressão arterial ao exercício. ^5 - 13^ No consenso *American College of Cardiology (ACC)/European Society of Cardiology (ESC)* 2003, a indicação de CDI era baseada no número de indicadores de risco. ^14^ Os critérios foram atualizados na diretriz *American College of Cardiology Foundation (ACCF)/American Heart Association (AHA)* 2011 em que foram incorporados fatores modificantes: mutações malignas, realce tardio à ressonância magnética (RM), aneurismas apicais e obstrução da via de saída do VE. ^15^ Novo modelo matemático-estatístico, adotado pela *ESC* em 2014, possibilita, com uma calculadora *online* , estimar o risco absoluto e a mortalidade em 5 anos por meio da atribuição de pesos aos quatro primeiros preditores independentes mencionados acrescidos por gradiente da via de saída do VE, diâmetro do átrio esquerdo e idade. ^16 , 17^

Em decorrência, o objetivo deste estudo é analisar o impacto do modelo *ESC* 2014 na determinação do risco de MS e indicação de CDI em prevenção primária em uma coorte de pacientes com CMH por meio da confrontação com os critérios prévios propostos por *ACCF/AHA* 2011.

## Métodos

### Seleção de pacientes

Foi analisada, retrospectivamente, uma coorte de 108 indivíduos registrados no ambulatório de CMH de um hospital universitário de atendimento terciário, entre março de 2007 e março de 2018. Os pacientes foram submetidos a eletrocardiograma em repouso, eletrocardiograma Holter e ecocardiograma, acrescidos por RM (n = 40; 45%). Estudo genético-molecular foi realizado em 18 (20%) indivíduos, cujos resultados foram previamente publicados. ^18^ O diagnóstico foi estabelecido de acordo com critérios definidos em diretrizes vigentes ^15 , 17^ com base na identificação de hipertrofia do VE detectada ao ecocardiograma e/ou RM pela presença de EPMVE ≥15 mm medida em qualquer segmento, com razão septo/parede posterior ≥1,3 na ausência de dilatação da câmara e outras condições capazes de produzir alterações semelhantes. Foram excluídos 18 casos por apresentarem período de seguimento <12 meses ou história prévia de parada cardiorrespiratória, fibrilação ventricular ou taquicardia ventricular com repercussão hemodinâmica. Os seguintes desfechos foram considerados para análise: 1) MS: fibrilação ventricular documentada, óbito até 1 hora do início de novos sintomas ou durante a noite sem agravamento clínico prévio; 2) choque apropriado pelo CDI por taquicardia ventricular ou fibrilação ventricular. O estudo foi aprovado pelo Comitê de Ética da instituição e desenvolvido de acordo com os princípios da Declaração de Helsinki. Termo de consentimento livre e esclarecido foi obtido de todos os pacientes.

### Estratificação de risco para morte súbita

Os seguintes preditores foram pesquisados: 1) idade; 2) história familiar de MS em primeiro grau, em <40 anos de idade ou em qualquer idade com diagnóstico confirmado da doença; 3) EPMVE medida ao ecocardiograma bidimensional; 4) síncope inexplicada nos últimos 6 meses; 5) TVNS definida por três ou mais batimentos extrassistólicos ventriculares sucessivos com frequência cardíaca ≥120 batimentos e duração <30s; 6) resposta anormal da pressão arterial ao exercício expressa pela incapacidade de elevar a pressão arterial sistólica ≥ 25 mmHg ou queda ≥10 mmHg no pico do exercício; 7) diâmetro do átrio esquerdo determinado ao ecocardiograma uni ou bidimensional; 8) gradiente máximo na via de saída do VE em repouso ou sob manobra de Valsalva com Doppler contínuo. Os seguintes modificantes de risco foram valorizados: 1) obstrução da via de saída do VE ≥30 mmHg; 2) realce tardio com gadolínio à RM cardíaca; 3) aneurisma apical do VE; 4) mutação genética maligna.

O escore *ESC HCM-Risk-SCD* para estimativa de risco de MS em 5 anos foi calculado pela seguinte fórmula:


Probabilidade em 5 anos = 1 – 0,998 exp (índice prognóstico)

$$\mathrm{Índice}\;\mathrm{prognóstico}\;=\;[0,15939858\;\times \;\mathrm{EPMVE}(\mathrm{mm})]\;–\;[0,00294271\;\times \;{\mathrm{EPMVE}}^{2}({\mathrm{mm}}^{2})]\;+\;[0,0259082\;\times \;\mathrm{diâmetro}\;\mathrm{do}\;\mathrm{átrio}\;\mathrm{esquerdo}(\mathrm{mm})]\;+\;[0,00446131\;x\;\mathrm{gradiente}\;\mathrm{máximo}\;\mathrm{na}\;\mathrm{via}\;\mathrm{de}\;\mathrm{saída}\;\mathrm{do}\;\mathrm{VE}\;(\mathrm{repouso}/\mathrm{Valsalva})(\mathrm{mmHg})]\;+\;[0,4583082\;\times \;\mathrm{história}\;\mathrm{familiar}\;\mathrm{de}\;\mathrm{MS}]\;+\;[0,82639195\;\times \;\mathrm{TVNS}]\;+\;[0,71650361\;\times \;\mathrm{síncope}\;\mathrm{inexplicada}]\;–\;[0,01799934\;\times \;\mathrm{idade}\;\mathrm{na}\;\mathrm{avaliação}(\mathrm{anos})].$$

Índice prognóstico = [0,15939858 × EPMVE(mm)] – [0,00294271 × EPMVE ^2^ (mm ^2^ )] + [0,0259082 × diâmetro do átrio esquerdo(mm)] + [0,00446131 x gradiente máximo na via de saída do VE (repouso/Valsalva)(mmHg)] + [0,4583082 × história familiar de MS] + [0,82639195 × TVNS] + [0,71650361 × síncope inexplicada] – [0,01799934 × idade na avaliação(anos)].

### Critérios de indicação de cardiodesfibrilador automático implantável

Os seguintes critérios para indicação de CDI em prevenção primária foram comparados:

1. Diretriz *ACCF* / *AHA* 2011: Classe IIa – história familiar de MS em 1º grau ou espessura parietal máxima do VE ≥30 mm ou síncope inexplicada recente; Classe IIa – TVNS ou resposta anormal da pressão arterial ao exercício associada a outros fatores ou modificantes de risco; Classe IIb – TVNS ou resposta anormal da pressão arterial ao exercício isoladas; Classe III – ausência dos fatores anteriormente citados.
2. Diretriz *ESC 2014* : Classe IIa – *HCM-Risk-SCD* ≥6%; Classe IIb – <6% e ≥4%; Classe III – <4%.

### Análise estatística

Os dados quantitativos foram apresentados por meio de média e desvio-padrão para distribuição normal ou por meio de medianas e intervalos interquartílicos (percentis 25 e 75) para distribuição não normal. A normalidade dos dados foi testada pelo teste Shapiro-Wilk. As variáveis categóricas foram descritas por meio de frequências absolutas e relativas. Variáveis contínuas com distribuição simétrica foram aferidas pelo teste *t* de Student para amostras independentes e análise de variâncias (ANOVA One-Way), variáveis categóricas pelos testes qui-quadrado e exato de Fisher e diferenças entre as categorias por meio dos resíduos ajustados. O coeficiente Kappa foi determinado para analisar a concordância entre as indicações para implante de CDI entre as diretrizes *ACCF* / *AHA* 2011 e *ESC* 2014. Os percentuais atingidos por cada um dos preditores que compõe o escore *ESC HCM-Risk-SCD* foram calculados pela média ponderada da variação de cada preditor na equação sobre a soma das variações desses preditores. A estimativa de sobrevida da amostra foi determinada por meio de curva de Kaplan-Meier. O tamanho da amostra foi estimado em 70 indivíduos para um valor esperado de Kappa = 0,3, se considerada a ocorrência de concordância entre as sistematizações, Kappa = 0, para poder de 90% e p < 0,05. Os dados foram processados no *software SPSS* versão 20.0 ( *SPSS Inc* ., Chicago, Illinois, EUA). O nível de significância adotado nas análises estatísticas foi de 5%.

## Resultados

### Características clínicas

A população em estudo foi constituída por 90 pacientes consecutivos com CMH, com idade média de 62±12 anos, 85 (94%) ≥40 anos e 56 (62%) do sexo feminino. As características clínicas da amostra encontram-se na Tabela 1 . No período médio de seguimento de 6±3 anos, 15 (17%) implantaram CDI para prevenção primária de MS. Dois (2%) pacientes apresentaram choque apropriado, 6 (7%) MS e 6 (7%) morte por outras causas ( Tabela 2 ).

**Tabela 1:** – Características clínicas de 90 pacientes com cardiomiopatia hipertrófica

Idade (anos)	62±12
Idade >40 anos (n,%)	85 (94%)
Sexo feminino (n,%)	56 (62%)
*Classe funcional NYHA*	
I/II (n,%)	75 (83%)
III/IV (n,%)	15 (17%)
Cardiopatia isquêmica (n,%)	11 (12%)
*Terapêutica*	
Betabloqueadores (n,%)	70 (78%)
Amiodarona (n,%)	20 (22%)
Verapamil/diltiazem (n,%)	24 (27%)
*Ecocardiograma*	
Diâmetro do AE (mm)	44±7
Diâmetro diastólico do VE (mm)	43±6
Diâmetro sistólico do VE (mm)	34±5
Espessura diastólica do septo (mm)	19±4
Espessura diastólica da parede posterior do VE (mm)	11±2
Fração de ejeção (%)	71±9
E/E´	16±8
Gradiente na via de saída do VE em repouso (mmHg)	28±31
Gradiente na via de saída do VE sob Valsalva (mmHg)	36±38
*Fatores de risco para MS*	
História familiar de MS*	23 (26%)
TVNS*	17 (19%)
Síncope*	16 (18%)
Resposta anormal da PA ao exercício*	9 (10%)
EPMVE >30 mm*	1 (1%)
Obstrução VSVE ≥30 mmHg ^†^	44 (49%)
Realce tardio à RM†	11 (12%)
Aneurisma apical de VE ^†^	0
Mutação maligna ^†^	0
*Número de fatores de risco*	
0	42 (47%)
1	32 (35%)
≥2	16 (18%)

*Preditores independentes; ^†^ Fatores modificantes; NYHA: New York Heart Association; AE: átrio esquerdo; VE: ventrículo esquerdo; MS: morte súbita; TVNS: taquicardia ventricular não sustentada; EPMVE: espessura parietal máxima do ventrículo esquerdo; PA: pressão arterial; VSVE: via de saída do ventrículo esquerdo; RM: ressonância magnética.

**Tabela 2:** – Dados evolutivos de 90 pacientes com cardiomiopatia hipertrófica seguidos por 6±3 anos

Insuficiência cardíaca Classe III/IV	20 (22%)
Fibrilação atrial (n,%)	29 (32%)
Ablação alcoólica do septo (n,%)	9 (10%)
Miectomia cirúrgica (n,%)	3 (3%)
Marca-passo (n,%)	6 (7%)
Implante de CDI (n,%)	15 (17%)
Choque apropriado CDI (n,%)	2 (2%)
Morte súbita (n,%)	6 (7%)
Morte por outras causas (n,%)	6 (7%)

*CDI: cardiodesfibrilador implantável.*

A sobrevida livre de MS ou choque apropriado pelo CDI no período de seguimento médio de 5 anos foi de 93% e, em 10 anos, de 92%. A sobrevida livre de morte por todas as causas em 5 e 10 anos foi de 80%.

### Estratificação de risco para morte súbita pelo escore *ESC HCM-risk SCD*

O escore *ESC HCM-risk-SCD* foi, em média, de 3,2±2,5% na amostra, estimado como baixo (<4%) em 67 (75%), médio (≥4% e <6%) em 11 (12%) e alto (>6%) em 12 (13%). A análise comparativa dos indicadores de MS valorizados nas duas normativas entre as três faixas de risco identificou que TVNS [3 (4%) *vs.* 6 (54%) *vs.* 8 (67%), p = 0,0001], síncope [6 (9%) *vs.* 3 (27%) *vs.* 7 (58%), p = 0,0001] e EPMVE mais elevada (17±3mm *vs.* 21±2mm *vs.* 21±8mm, p = 0,002) foram predominantes entre aqueles com maior predisposição. Os demais preditores não diferiram entre os grupos ( Tabela 3 ). Choque apropriado pelo CDI ou MS foi semelhante entre os pacientes das faixas de baixo, médio e alto risco [6 (8,8%) *vs.* 2 (18,2%) *vs.* 0 (0%), p = 0,22].

**Tabela 3:** – Distribuição dos preditores de morte súbita nas três faixas de risco da diretriz *European Society of Cardiology* 2014

	Risco de MS			p

	<4%	≥4%-<6	≥6%	
	(n = 67; 75%)	(n = 11; 12%)	(n = 12; 13%)	
Idade (anos)	64±11	60±17	57±13	0,156
História familiar de MS	14 (21%)	4 (36%)	5 (42%)	0,177
Síncope	6 (9%)	3 (27%)	7 (58%)	0,0001
EPMVE ≥30 mm	0	0	1	0,264
TVNS	3 (4%)	6 (54%)	8 (67%)	0,0001
Resposta anormal da PA ao exercício	8 (12%)	0	1 (8%)	0,595
Realce tardio à ressonância magnética	8 (12%)	1 (9%)	2 (17%)	0,822
Obstrução VSVE ≥30 mmHg	31 (46%)	7 (64%)	6 (50%)	0,649
Diâmetro do átrio esquerdo (mm)	46±7	48±9	48±8	0,545
Espessura parietal máxima do VE (mm)	17±3	21±2	21±8	0,002
Gradiente máximo VSVE (mmHg)	33±42	45±39	40±44	0,77

*MS: morte súbita; EPMVE: espessura parietal máxima do ventrículo esquerdo; TVNS: taquicardia ventricular não sustentada; PA: pressão arterial; VSVE: via de saída do ventrículo esquerdo; VE: ventrículo esquerdo.*

A Tabela 4 apresenta os percentuais atingidos por cada um dos preditores que compõe o escore *ESC HCM-Risk-SCD* nas três categorias de risco para MS. Os fatores que mais contribuíram para o cálculo do escore nas faixas de baixo, médio e alto risco foram EPMVE, diâmetro do átrio esquerdo e idade. Gradiente na via de saída do VE, história familiar de MS, TVNS e síncope evidenciaram menor contribuição.

**Tabela 4:** – Contribuição dos preditores de morte súbita para o cálculo do *escore ESC HCM-Risk-SCD*

	Baixo risco		Médio risco		Alto risco	

	<4%		≥4% - <6%		≥ 6%	
	Mediana	p25 - p75	Mediana	p25 - p75	Mediana	p25 - p75
*ESC HCM-Risk-SCD*	1,88%	1,42 - 2,67	5,17%	4,89 - 5,70	7,82%	7,06 - 9,19
Espessura parietal máxima do VE	1,60%	1,25 - 2,02	3,20%	3,18 - 3,36	4,46%	4,07 - 5,09
Diâmetro do AE	0,97%	0,83 - 1,21	1,86%	1,67 - 2,40	2,48%	2,21 - 3,51
Gradiente na via de saída do VE	0,03%	0,01 - 0,24	0,34%	0,15 - 0,61	0,35%	0,02 - 1,00
História familiar de MS	0,00%	0,00 - 0,00	0,00%	0,00 - 0,70	0,00%	0,00 - 0,99
TVNS	0,00%	0,00 - 0,00	1,14%	0,00 - 1,30	1,64%	0,00 - 1,96
Síncope	0,00%	0,00 - 0,00	0,00%	0,00 - 1,09	1,41%	0,00 - 1,59
Idade	-0,91%	0,8 - 1,13	-1,90%	1,12 - 2,03	-2,34%	1,49 - 2,73

*ESC: European Society of Cardiology; VE: ventrículo esquerdo; AE: átrio esquerdo; MS: morte súbita; TVNS: taquicardia ventricular não sustentada.*

### Comparação das diretrizes *American College of Cardiology Foundation/American Heart Association* 2011 e *European Society of Cardiology* 2014

**Figura 1:**
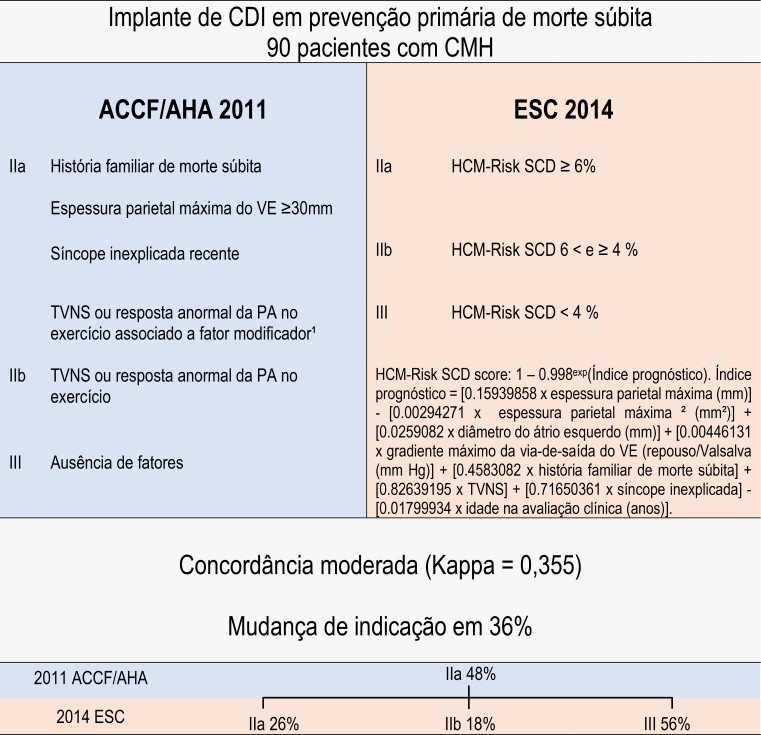
– *Discordância entre diretrizes ACCF/AHA 2011 e ESC 2014 sobre critérios de prevenção primária de morte súbita na cardiomiopatia hipertrófica* CMH: cardiomiopatia hipertrófica, CDI = cardiodesfibrilador implantável, ACCF= American College of Cardiology Foundation, AHA = American Heart Association, ESC = European Society of Cardiology, VE=ventrículo esquerdo, TVNS, taquicardia ventricular não-sustentada, PA=pressão arterial. ^1^ Fator modificador: 1. Obstrução da via de saída do VE ≥30 mmHg; 2. Realce tardio com gadolíneo à ressonância magnética cardíaca; 3. Aneurisma apical do VE; 4. Mutação genética maligna.

De acordo com os critérios *ACCF/AHA* 2011, 43 (48%) pacientes receberam indicação de CDI Classe IIa, três (3%) Classe IIb e 44 (49%) Classe III. Com base na diretriz *ESC* 2014, 12 (14%) pacientes constituíram recomendação Classe IIa, 11 (12%) Classe IIb e 67 (74%) Classe III. A comparação dos graus de indicação de CDI entre as duas sistematizações revelou baixa concordância (Kappa = 0,355, p = 0,0001). O escore *ESC HCM-risk-SCD* reduziu a classe de recomendação de CDI em 32 (36%) pacientes, manteve em 57 (63%) e acrescentou *status* para implante em somente um (1%). Dos 43 (48%) indivíduos que receberam indicação IIa pela *ACCF/AHA 2011* , 32 (74%) tiveram a recomendação reduzida pela *ESC 2014* , 8 (18%) para IIb e 24 (56%) para III. Apenas 11(26%) conservaram a indicação Classe IIa. Dos 44 pacientes (49%) em Classe III pela *ACCF/AHA* 2011, 43 (98%) mantiveram a contraindicação pela sistematização europeia ( Tabela 5 ). A Figura 1 apresenta o resumo do estudo e seus principais resultados.

**Tabela 5:** – Comparação das indicações de cardiodesfibrilador implantável entre as diretrizes *American College of Cardiology Foundation/American Heart Association* 2011 e *European Society of Cardiology* 2014

			*ESC* 2014		
			IIa	IIb	III
		n (%)	12 (14%)	11 (12%)	67 (74%)
*ACCF/AHA 2011*	IIa	43 (48%)	11 (26%)	8 (18%)	24 (56%)
	IIb	3 (3%)	0	3 (100%)	0
	III	44 (49%)	1 (2%)	0	43 (98%)
		Kappa = 0,355, P = 0,0001			

*ACCF/AHA: American College of Cardiology Foundation/American Heart Association; ESC: European Society of Cardiology.*

O escore *ESC* foi de 3±1,7% nos 8 (9%) pacientes que sofreram MS ou choque apropriado. Quatro (50%) apresentavam indicação Classe IIa para implante do dispositivo pela *ACCF/AHA* 2011, mas nenhum atingiu esse grau de recomendação pela *ESC* 2014 *,* ainda que 2 (25%) pacientes tenham permanecido em Classe IIb.

Os fatores de risco que na amostra caracterizaram recomendação IIa pela *ACCF/AHA 2011* conjuntamente associaram-se à perda de indicação na reestratificação pela *ESC* (p = 0,05). História familiar de MS e TVNS adicionada à obstrução da via de saída de VE foram os preditores que apresentaram maior perda de recomendação de CDI pela diretriz europeia ( Tabela 6 ).

**Tabela 6:** – Perfil de risco para morte súbita em pacientes com cardiomiopatia hipertrófica e indicação Classe IIa de cardiodesfibrilador implantável pela *American College of Cardiology Foundation/American Heart Association* 2011: reestratificação de risco pela *European Society of Cardiology* 2014

	*ACCF/AHA* 2011/ *ESC* 2014		

	IIa 2011/IIa 2014	IIa 2011/IIb 2014	IIa 2011/III 2014
	n = 11 (26%)	n = 8 (19%)	n = 24 (55%)
HFMS isolada	2 (12%)	2 (12%)	13 (76%)
Síncope isolada	5 (45%)	1 (10%)	5 (45%)
Síncope + HFMS	2 (40%)	2 (40%)	1 (20%)
HFMS + EPMVE ≥30mm	1 (100%)	0	0
TVNS + obstrução VSVE	1 (17%)	3 (50%)	2 (33%)
Resposta anormal PA + obstrução VSVE + realce tardio	0	0	3 (100%)
	P = 0,05		

*HFMS: história familiar de morte súbita; EPMVE: espessura parietal máxima do ventrículo esquerdo; TVNS: taquicardia ventricular não sustentada; VSVE: via de saída do ventrículo esquerdo; PA: pressão arterial; ACCF: American College of Cardiology Foundation. AHA: American Heart Association; ESC: European Society of Cardiology.*

## Discussão

Neste estudo, confrontamos, pela primeira vez, os critérios para prevenção primária de MS propostos pela *ACCF/AHA* 2011 e *ESC* 2014 em uma população brasileira de portadores de CMH com base em uma coorte de pacientes ambulatoriais não referenciados. Nossos resultados demonstram baixa concordância entre as duas sistematizações quanto aos critérios de indicação de CDI em prevenção primária. O escore *ESC HCM-Risk-SCD* reduziu a classe de recomendação para implante em relação a *ACCF/AHA* em 36% dos pacientes. Entre aqueles situados em Classe IIa pela diretriz norte-americana, 74% tiveram o grau de indicação reduzido pela *ESC* e 56% configuraram contraindicação para implante de CDI, sendo a recomendação mantida em apenas 26%. O escore europeu acrescentou indicação em somente 1% dos pacientes. Na quase totalidade dos casos em que o implante não foi recomendado pela diretriz norte-americana, a contraindicação foi mantida pelos critérios da *ESC* . O novo modelo excluiria da Classe IIa os 8 (9%) pacientes que sofreram MS ou choque apropriado pelo CDI durante o período de observação, ainda que 25% desses tenham permanecido em Classe IIb.

A CMH é uma cardiopatia de caráter arritmogênico, cujo substrato histopatológico constituído por hipertrofia, desorganização celular, fibrose e doença da microcirculação favorece o desenvolvimento de arritmias ventriculares fatais. ^5 , 6 , 19 , 20^ A estratificação de risco para MS baseia-se em dados observacionais coletados de populações muito selecionadas. É considerada complexa em decorrência do caráter heterogêneo da doença e julgada ainda imperfeita pelo fato de muitos óbitos ocorrerem na ausência de preditores. ^5 - 7 , 21^ A limitação oferecida pelos algoritmos de 2003 e 2011 foi demonstrada em um registro internacional que evidenciou ausência de diferença nas taxas de choques apropriados entre os pacientes com um, dois, três ou mais predisponentes. ^22^ Análise posterior de validação desses critérios verificou que a incidência de MS e choque apropriado não divergiu entre pacientes com um ou nenhum preditor e que os algoritmos iniciais teriam poder limitado para discriminar indivíduos com maior ou menor predisposição e implicariam em número substancial de implantes desnecessários. ^23^

No presente estudo, foi avaliada uma coorte de CMH com faixa etária mais avançada e características de baixo risco: 78% mantiveram-se em classe funcional I/II, 47% não apresentavam fatores predisponentes para MS e 35% evidenciavam apenas um. Portadores de CMH ≥60 anos de idade exibem taxas mais reduzidas de morbimortalidade e MS, mesmo na presença de preditores. ^6^ A sobrevida livre de MS ou choque apropriado pelo CDI atingiu 93% em 5 anos e 92% em 10 anos, sendo que, no período, apenas 9% dos indivíduos desenvolveram esses eventos. Estudo longitudinal, multicêntrico, demonstra resultados semelhantes e destaca que a CMH, quando convenientemente tratada, apresenta reduzida mortalidade na fase adulta, com sobrevida média em 10 anos semelhante à esperada na população em geral. ^3^

O escore *ESC HCM-risk-SCD* , de 3,2±2,5%, caracterizou risco reduzido de MS em 75% dos pacientes. TVNS, síncope e maior EPMVE foram mais frequentes na faixa de alto risco em relação às demais. Nesse estudo, determinamos os percentuais atingidos por cada um dos preditores integrantes do escore nas três categorias de risco com objetivo de discriminar aqueles que atingiram maior peso e que pudessem justificar a baixa concordância entre as duas diretrizes. Verificou-se que os fatores que mais contribuíram para o cálculo, por terem alcançado valores mais elevados nas faixas de baixo, médio e alto risco, foram EPMVE, diâmetro do átrio esquerdo e idade, a qual tem efeito subtrativo. Esses achados podem justificar a baixa concordância entre as duas diretrizes, visto que EPMVE como variável contínua, diâmetro do átrio esquerdo e idade não constam da normativa norte-americana. História familiar de MS e síncope isoladas, que na *ACCF/AHA* constituem indicação para implante, evidenciaram menor contribuição para o cálculo do escore.

Os fatores de risco que na amostra caracterizaram indicação IIa para CDI pela *ACCF/AHA* , notadamente, história familiar de MS e TVNS adicionada à obstrução da via de saída do VE, associaram-se à perda de recomendação pela *ESC,* atingindo *status* de contraindicação em 55% dos casos. Nossos resultados sugerem que a perda de recomendação para implante de CDI proporcionada pelo escore europeu se relaciona sobretudo a situações em que a indicação pela diretriz norte-americana está fundamentada na presença de um preditor isolado ou em associação a fatores modificantes. Esses achados são justificados pelo fato de que o modelo europeu define prevenção primária com base no conjunto de fatores de risco e não na presença de um só indicador.

O escore *ESC HCM-Risk-SCD* foi validado de modo independente em populações de três continentes em estudos observacionais que, em sua maioria, demonstram que o novo modelo contribui para o aprimoramento da estratificação de risco e do processo decisório na CMH. ^24 - 28^ Outras análises assinalam a pouca sensibilidade do escore para o reconhecimento dos pacientes mais suscetíveis, a maior capacidade de identificar casos sem indicação de CDI e o registro de taxas similares de eventos nas três faixas de risco. ^29 - 32^ Nosso estudo corrobora esses achados ao demonstrar que o modelo europeu reduz as recomendações de CDI em relação à diretriz norte-americana, deixa desprotegida a totalidade de pacientes que sofreram MS ou choque apropriado e estabelece maior concordância nos casos de contraindicação de implante do dispositivo. Contudo, a metanálise de seis estudos, com 7.291 pacientes, demonstra que, na maioria dos casos, a suscetibilidade à MS em 5 anos foi adequadamente estimada pelo escore ESC. ^33^

O escore europeu fundamenta a estratificação de risco para MS por meio da aplicação de um rígido modelo matemático-estatístico em uma doença complexa de evolução imprevisível. Limitações metodológicas podem existir na dependência da avaliação do átrio esquerdo pelo respectivo diâmetro, da obstrução da via de saída pela manobra de Valsalva e pela exclusão de isquemia miocárdica, realce tardio e aneurismas apicais do VE. Embora restrições possam ser admitidas em relação ao seu desempenho, particularmente no alto risco, o escore europeu merece assimilação na prática clínica como forma validada de orientar decisões terapêuticas. A determinação dos percentuais atingidos pelos preditores integrantes da fórmula caso a caso poderá contribuir para a interpretação de resultados na prática clínica. Na presente análise, a normativa norte-americana asseguraria proteção a um número maior de indivíduos em relação ao critério europeu. Todavia, poderia resultar em implantes desnecessários e exporia essa população a complicações inerentes ao dispositivo, tais como infecções e choques inapropriados. ^4 , 15 , 17^ São necessários estudos prospectivos validados igualmente em populações de menor risco, com o objetivo de possibilitar a identificação de novos fatores predisponentes e o refinamento dos critérios de indicação de CDI para prevenção primária de MS nessa doença.

### Limitações do estudo

O presente estudo baseia-se na avaliação de uma coorte bem documentada de CMH de um único centro com menor grau de seleção e faixa etária mais avançada. As características clínicas e as reduzidas taxas de eventos indicam tratar-se de pacientes de menor risco, distintos daqueles que integram a maioria dos estudos de validação. Esses aspectos podem limitar as conclusões a populações com esse perfil. Contudo, os casos incluídos são tão representativos da doença quanto aqueles de maior risco e mais suscetíveis a complicações, selecionados em centros de referência.

## Conclusão

No estudo de uma coorte de CMH de menor risco e faixa etária mais avançada, baixa concordância foi identificada entre os critérios de prevenção primária de MS estabelecidos pelas diretrizes *ACCF/AHA 2011 e ESC 2014.* O escore *ESC HCM-Risk-SCD* reduziu as indicações para implante de CDI na população estudada, notadamente naqueles situados em Classe IIa na sistematização norte-americana, mas deixou desprotegida a totalidade de pacientes que sofreram MS ou choque apropriado. A maior contribuição para o cálculo do escore de preditores não incluídos na normativa *ACCF/AHA 2011* poderia justificar, em parte, a discordância entre as duas diretrizes.
