# Supplementary material for: Discordância entre Diretrizes Internacionais sobre Critérios de Prevenção Primária de Morte Súbita na Cardiomiopatia Hipertrófica
Source: Arq Bras Cardiol. 2020 Aug 19;115(2):197–204. [Article in Portuguese] doi: 10.36660/abc.20190161 (PMC8384276; doi:10.36660/abc.20190161)
Supplement: Translation

# Translation: Discrepancy between International Guidelines on the Criteria for Primary Prevention of Sudden Cardiac Death in Hypertrophic Cardiomyopathy

**Authors:** Beatriz Piva e Mattos, Fernando Luís Scolari, Henrique Iahnke Garbin

**Affiliations:** 1 Brazil Hospital de Clínicas de Porto Alegre, Porto Alegre, RS - Brazil; 2 Brazil Universidade Federal do Rio Grande do Sul - Faculdade de Medicina, Porto Alegre, RS - Brazil

**Keywords:** Cardiomyopathy,Hypertrophic/genetics, Heredity, Death,Sudden, Cardiac, Arrhythmias, Cardiac, Syncope, Defibrillators,Implantable, Cohort Studies

## Abstract

**Background:**

Risk stratification for sudden cardiac death (SCD) in hypertrophic cardiomyopathy (HCM) is based on different algorithms proposed by the 2011 ACCF/AHA and 2014 ESC guidelines.

**Objective:**

To analyze the 2014 ESC model for SCD risk stratification and primary prevention ICD (implantable cardioverter defibrillator) in HCM in comparison to the North American guideline.

**Methods:**

An HCM cohort was evaluated and the ESC HCM-Risk SCD score was calculated. Agreement of ICD recommendations criteria between the two guidelines was analyzed with Kappa coefficient. P<0.05 was adopted for the statistical analysis.

**Results:**

In 90 consecutive patients followed for 6±3 years, the mean calculated ESC risk score was 3.2±2.5%. The risk predictors that have mainly contributed to the score calculation in the low (1.88% [1.42–2.67]), intermediate (5.17% [4.89–5.70]) and high-risk (7.82% [7.06–9.19]) categories were: maximal left ventricular wall thickness (1.60% [1.25–2.02]; 3.20% [3.18–3.36]; 4.46% [4.07–5.09]), left atrial diameter (0.97% [0.83–1.21]; 1.86% [1.67-2.40]; 2.48% [2.21–3.51]) and age (-0.91% [0.8–1.13]; -1.90% [1.12–2.03]; -2.34% [1.49–2.73]). The European model decreased the ICD recommendations in 32 (36%) patients. Among the 43 (48%) individuals with class IIa recommendation under the 2011 ACCF/AHA guideline, 8 (18%) were downgraded to class IIb and 24 (56%) to class III. Low agreement was found between the two systems: Kappa=0.355 and p=0.0001. In 8 (9%) patients with SCD or appropriate shock, 4 (50%) met class IIa indication with the 2011 ACCF/AHA guideline, but none achieved this class of recommendation with the 2014 ESC model.

**Conclusion:**

Low agreement was found between the two strategies. The novel ESC model decreased the ICD recommendations, especially in those with class IIa recommendation, but left unprotected all patients with SCD or appropriate shock. (Arq Bras Cardiol. 2020; 115(2):197-204)

## Introduction

Hypertrophic cardiomyopathy (HCM) represents the most prevalent form of genetic heart disease, affecting one in 200 individuals.^1^ Sudden cardiac death (SCD), presently estimated at 0.5 to 1%/year, occurs at any age, although it predominates in young subjects and athletes.^2 - 4^

The risk stratification for SCD is the basis for the recommendation of implantable cardioverter defibrillator (ICD) in HCM, the only approach considered to be able to modify the disease prognosis.^4 - 7^ There is a consensus about the recommendation in patients with prior cardiac arrest. However, many questions persist regarding primary prevention. Five risk factors identified in longitudinal studies and validated in meta-analyses are recognized as independent predictors of SCD: family history, unexplained syncope, maximal left ventricular wall thickness (MLVWT) ≥30 mm, non-sustained ventricular tachycardia (NSVT) and abnormal blood pressure response to exercise.^5 - 13^ In the 2003 American College of Cardiology (ACC)/European Society of Cardiology (ESC) Consensus, the ICD recommendation was based on the number of risk markers.^14^ The criteria were updated in the 2011 American College of Cardiology Foundation (ACCF)/American Heart Association (AHA) guideline, in which modifying factors were included: malignant mutations, late gadolinium enhancement, left ventricular (LV) apical aneurysms and outflow tract obstruction.^15^ A novel mathematical and statistical prediction model endorsed by ESC in 2014 and accessible with an online calculator provides an estimate of the absolute risk and five-year mortality rate, applying different weights to the former five independent predictors mentioned above, in addition to LV outflow gradient, left atrial diameter and age.^16 , 17^

The purpose of the study is to analyze the impact of the 2014 ESC model on the SCD risk stratification and the recommendations of ICD primary prevention compared to the previously proposed 2011 ACCF/AHA criteria.

## Methods

### Patient selection

A cohort of 108 subjects followed at a dedicated HCM outpatient clinic of a tertiary center from March 2007 to March 2018 was retrospectively studied. All patients were submitted to rest electrocardiogram, 24-hour Holter electrocardiogram and echocardiogram. Cardiac magnetic resonance (CMR) imaging was applied to 40 (45%) subjects. Molecular-genetic testing was performed in 18 (20%) patients, whose results were previously published.^18^ Diagnosis was established according to the current guidelines^15 , 17^ based on the identification of unexplained LV hypertrophy detected on echocardiogram and/or magnetic resonance imaging by the presence of MLVWT ≥15 mm measured at any segment, with septum/posterior wall ratio ≥1.3 in the absence of chamber dilation or other conditions capable of producing a similar pattern of hypertrophy. Eighteen cases were excluded due to a follow-up period <12 months or previous history of cardiopulmonary arrest, ventricular fibrillation or ventricular tachycardia with hemodynamic impairment. The following outcomes were considered for the analysis: 1. Sudden cardiac death: documented ventricular fibrillation, death one hour from the onset of symptoms or at night without previous clinical worsening; 2. Appropriate ICD shock for ventricular tachycardia or ventricular fibrillation. The study was approved by the Ethics Committee of the institution and performed under the principles of the principles of the Declaration of Helsinki. Written informed consent was obtained from all patients.

### Risk stratification for sudden cardiac death

The following predictors were assessed: 1. Age; 2. Family history of SCD in first-degree relatives, <40 years old or at any age with previous diagnosis of the disease; 2. MLVWT measured on echocardiogram; 4. Unexplained syncope within the past 6 months; 5. NSVT defined as three or more successive premature ventricular beats at a heart rate ≥120 beats/min lasting ≤30 s; 6. Abnormal blood pressure response to exercise defined as <25 mmHg rise and/or 10 mmHg drop of maximal systolic blood pressure during peak exercise; 7. Left atrial diameter obtained on M-mode or two-dimensional echocardiogram; 8. Maximum left ventricular outflow tract (LVOT) gradient at rest or with Valsalva maneuver using continuous wave Doppler. The following risk modifiers were considered: 1. LVOT gradient ≥ 30 mmHg; 2. Late gadolinium enhancement on CMR; 3. LV apical aneurysm; 4. Malignant genetic mutations.

The probability of SCD in 5 years was calculated with the ESC HCM-Risk SCD equation as follows:


$$\mathrm{Probability}\;\mathrm{of}\;\mathrm{SCD}\;\mathrm{in}\;5\;\mathrm{years}\;=\;1\;–\;0.998^{\exp(\mathrm{Prognostic}\;\mathrm{index})}$$

$$\mathrm{Prognostic}\;\mathrm{index}\;=\;[0.15939858\;x\;\mathrm{maximal}\;\mathrm{wall}\;\mathrm{thickness}\;(\mathrm{mm})]\;-\;[0.00294271\;x\;\mathrm{maximal}\;\mathrm{wall}\;{\mathrm{thickness}}^{2}({\mathrm{mm}}^{2})]\;+\;[0.0259082\;x\;\mathrm{left}\;\mathrm{atrial}\;\mathrm{diameter}\;(\mathrm{mm})]\;+\;[0.00446131\;x\;\mathrm{maximal}\;(\mathrm{rest}/\mathrm{Valsalva})\;\mathrm{LVOT}\;\mathrm{gradient}\;(\mathrm{mm}\;\mathrm{Hg})]\;+\;[0.4583082\;x\;\mathrm{family}\;\mathrm{history}\;\mathrm{SCD}]\;+\;[0.82639195\;x\;\mathrm{NSVT}]\;+\;[0.71650361\;x\;\mathrm{unexplained}\;\mathrm{syncope}]\;-\;[0.01799934\;x\;\mathrm{age}\;\mathrm{at}\;\mathrm{clinical}\;\mathrm{evaluation}\;(\mathrm{years})].$$

Prognostic index = [0.15939858 x maximal wall thickness (mm)] - [0.00294271 x maximal wall thickness^2^(mm^2^)] + [0.0259082 x left atrial diameter (mm)] + [0.00446131 x maximal (rest/Valsalva) LVOT gradient (mm Hg)] + [0.4583082 x family history SCD] + [0.82639195 x NSVT] + [0.71650361 x unexplained syncope] - [0.01799934 x age at clinical evaluation (years)].

### Recommendations of implantable cardioverter defibrillator therapy

The following criteria for primary prevention ICD were compared:

1. 2011 ACCF/AHA guideline: Class IIa - A family history of SCD in a first-degree relative or MLVWT ≥ 30 mm or unexplained syncope. Class IIa - NSVT or abnormal blood pressure response to exercise associated with other risk factors or modifiers; Class IIb: Isolated NSVT or abnormal blood pressure response to exercise; Class III – absence of the previously mentioned risk factors.
2. 2014 ESC guideline: Class IIa – HCM Risk-SCD ≥6%; Class IIb - <6% and ≥4%; Class III - <4%.

### Statistical analysis

Normally distributed continuous variables were expressed as mean±standard deviation and non-normally distributed data presented as median and interquartile ranges (percentile 25 and 85). Continuous variables were tested for normality using the Shapiro-Wilk test. Categorical variables were described as absolute and relative frequencies. Continuous variables were compared with Student’s *t* test or one-way analysis of variance (ANOVA), categorical variables with chi-square or Fisher’s exact test and differences among categories with standardized adjusted residual analysis. The Kappa coefficient was calculated to determine the agreement between the 2011 ACCF/AHA and the 2014 ESC guidelines for primary prevention ICD. The percentages achieved by each of the risk predictors included in the ESC HCM Risk-SCD score were calculated with the weighted average of the variation of each predictor in the equation over the sum of the variations of these predictors. The estimated survival of the sample was determined using the Kaplan-Meier curve. The sample size was estimated at 70 individuals for an expected Kappa=0.3, considering the occurrence of agreement between the guidelines, Kappa=0 for 90% power and p<0.05. SPSS software version 20.0 (SPSS Inc., Chicago, Illinois, USA) was used for the analyses. All comparisons were two-tailed and p<0.05 was considered to be statistically significant.

## Results

### Clinical characteristics

The study population comprised 90 consecutive patients with HCM, mean age 62±12 years, 85 (94%) ≥40 years and 56 (62%) females. The clinical characteristics of the study patients are described in table 1 . Along the follow-up period of 6±3 years, 15 (17%) patients received an ICD for SCD primary prevention. Two (2%) patients experienced appropriate shock, 6 (7%) experienced SCD and 6 (7%) had death unrelated to HCM ( Table 2 ).

**Table 1:** – Clinical characteristics of 90 patients with hypertrophic cardiomyopathy

Age (years)	62±12
Age >40 years (n, %)	85 (94%)
Female sex (n, %)	56 (62%)
*NYHA functional class*	
I/II (n, %)	75 (83%)
III/IV (n, %)	15 (17%)
Coronary artery disease (n, %)	11 (12%)
*Treatment*	
Betablockers (n, %)	70 (78%)
Amiodarone (n, %)	20 (22%)
Verapamil/diltiazem (n, %)	24 (27%)
*Echocardiogram*	
LA diameter (mm)	44±7
LV diastolic diameter (mm)	43±6
LV systolic diameter (mm)	34±5
Septal diastolic thickness (mm)	19±4
LV posterior wall diastolic thickness (mm)	11±2
Ejection fraction (%)	71±9
E/E´	16±8
LVOT gradient at rest (mmHg)	28±31
LVOT gradient with Valsalva maneuver (mmHg)	36±38
*SCD risk factors*	
Family history of SCD*	23 (26%)
NSVT*	17 (19%)
Syncope*	16 (18%)
Abnormal BP response to exercise *	9 (10%)
MLVWT >30 mm*	1 (1%)
LVOT gradient ≥30 mmHg^†^	44 (49%)
LGE on CMR^†^	11 (12%)
LV apical aneurysm^†^	0
Malignant mutation^†^	0
*Number of SCD risk factors*	
0	42 (47%)
1	32 (35%)
≥2	16 (18%)

*Independent predictors^†^Modifying factors; NYHA: New York Heart Association; LA: left atrium; LV: left ventricle; LVOT: left ventricular outflow tract; SCD: sudden cardiac death; NSVT: non-sustained ventricular tachycardia; BP: blood pressure; MLVWT: left ventricle maximal wall thickness; LGE: late gadolinium enhancement; CMR; cardiac magnetic resonance.

**Table 2:** – Cardiovascular outcomes in 90 patients with hypertrophic cardiomyopathy patients during a 6±3-year follow-up

Heart failure class III/IV	20 (22%)
Atrial fibrillation (n, %)	29 (32%)
Alcohol septal ablation (n, %)	9 (10%)
Surgical myectomy (n, %)	3 (3%)
Dual-chamber pacemaker (n, %)	6 (7%)
ICD implantation (n, %)	15 (17%)
Appropriate ICD shock (n, %)	2 (2%)
Sudden cardiac death (n, %)	6 (7%)
HCM non-related death (n, %)	6 (7%)

*ICD: implantable cardioverter defibrillator; HC:= hypertrophic cardiomyopathy.*

Five and ten-year SCD or ICD appropriate shock free survival rates were 93% and 92%, respectively, in the period. Five and ten-year all-cause death-free survival in five and ten years was 80%.

### ESC HCM risk-SCD score for sudden cardiac death risk stratification

The mean calculated ESC HCM risk-SCD was 3.2±2.5% in the sample and it was estimated as low (<4%) in 67 (75%) patients, intermediate (≥4%–<6%) in 11 (12%) and high (>6%) in 12 (13%). The comparative analysis of SCD markers adopted in the two guidelines between the three risk ranges showed that NSVT [3 (4%) vs. 6 (54%) vs. 8 (67%), p=0.0001], syncope [6 (9%) vs. 3 (27%) vs. 7 (58%), p=0.0001] and increased MLVWT (17±3mm vs. 21±2mm vs. 21±8 mm, p=0.002) were predominant in higher risk. The other predictors do not differ between the groups ( Table 3 ). SCD or appropriate ICD shock rates were similar between low, medium and high-risk patients [6 (8.8%) vs. 2 (18.2%) vs. 0 (0%), p=0.22].

**Table 3:** – Distribution of sudden cardiac death predictors in the three risk categories of the 2014 European Society of Cardiology guideline

	ESC HCM Risk-SCD score			p

	<4%	≥4%-<6	≥6%	
	(n=67;75%)	(n=11;12%)	(n=12;13%)	
Age (years)	64±11	60±17	57±13	0.156
Family history of SCD	14(21%)	4(36%)	5(42%)	0.177
Syncope	6(9%)	3(27%)	7(58%)	0.0001
MLVWT ≥30 mm	0	0	1	0.264
NSVT	3(4%)	6(54%)	8(67%)	0.0001
Abnormal BP response to exercise	8(12%)	0	1(8%)	0.595
LGE on CMR	8(12%)	1(9%)	2(17%)	0.822
LVOT ≥30 mmHg	31(46%)	7 (64%)	6(50%)	0.649
Left atrial diameter (mm)	46±7	48±9	48±8	0.545
MLVWT (mm)	17±3	21±2	21±8	0.002
Maximal LVOT gradient (mmHg)	33±42	45±39	40±44	0.77

*SCD: sudden cardiac death, MLVWT: maximal left ventricular wall thickness; NSVT: non-sustained ventricular tachycardia; BP: blood pressure; LGE: late gadolinium enhancement; CMR; cardiac magnetic resonance; LVOT: left ventricular outflow tract.*

Table 4 presents the percentages achieved by each of the variables included in the ESC HCM Risk-SCD score in the three risk categories. The risk factors that have mainly contributed to the score calculation in the low, intermediate and high-risk levels were MLVWT, left atrial diameter and age. LVOT obstruction, family history of SCD, NSVT and syncope reached lower weights.

**Table 4:** – Contribution of sudden cardiac death risk predictors for the ESC HCM Risk-SCD score calculation

	Low risk		Intermediate risk		High risk	

	<4%		≥4% - <6%		≥ 6%	
	Median	p25 - p75	Median	p25 - p75	Median	p25 - p75
ESC HCM Risk-SCD	1.88%	1.42-2.67	5.17%	4.89-5.70	7.82%	7.06-9.19
MLVWT	1.60%	1.25-2.02	3.20%	3.18-3.36	4.46%	4.07-5.09
Left atrial diameter	0.97%	0.83-1.21	1.86%	1.67-2.40	2.48%	2.21-3.51
LVOT gradient	0.03%	0.01-0.24	0.34%	0.15-0.61	0.35%	0.02-1.00
Family history of SCD	0.00%	0.00-0.00	0.00%	0.00-0.70	0.00%	0.00-0.99
NSVT	0.00%	0.00-0.00	1.14%	0.00-1.30	1.64%	0.00-1.96
Syncope	0.00%	0.00-0.00	0.00%	0.00-1.09	1.41%	0.00-1.59
Age	-0.91%	0.8 - 1.13	-1.90%	1.12-2.03	-2.34%	1.49-2.73

*ESC: European Society of Cardiology; MLVWT: maximal left ventricular wall thickness; LVOT: left ventricular outflow tract; SCD: sudden cardiac death; NSVT: non-sustained ventricular tachycardia.*

Comparison between the 2011 American College of Cardiology Foundation/American Heart Association and the 2014 European Society of Cardiology guidelines

According to the 2011 ACCF/AHA criteria, 43 (48%) patients received class IIa recommendation for ICD, 3 (3%) class IIb and 44 (49%) class III. In the 2014 ESC guideline, 12 (14%) patients received class IIa recommendation for ICD therapy, 11 (12%) class IIb, and 67 (74%) class III. Comparison of the classes of ICD recommendations showed low agreement (Kappa=0.355, p=0.0001) between the two guidelines. The ESC HCM risk-SCD score decreased the ICD recommendations in 32 (36%) patients, maintained in 57 (63%) and provided an additional recommendation in only one (1%). Of the 43 (48%) individuals with class IIa recommendation under the 2011 ACCF/AHA guideline, the ESC risk score decreased the class of recommendation for ICD therapy in 32 (74%) patients, 8 (18%) for class IIb and 24 (56%) for class III. Only 11 (26%) remained in class IIa recommendation. Of the 44 (49%) patients in class III with the 2011 ACCF/AHA guideline, the European model determined an ICD unwarranted in 43 (98%) ( Table 5 ). Figure 1 shows the study summary and its main findings.

**Table 5:** – Comparison of implantable cardioverter defibrillator recommendations between the 2011 American College of Cardiology Foundation/American Heart Association and the 2014 European Society of Cardiology guidelines

			2014 ESC		

			IIa	IIb	III
		n (%)	12 (14%)	11 (12%)	67 (74%)
2011 ACCF/AHA	IIa	43(48%)	11(26%)	8(18%)	24(56%)
	IIb	3(3%)	0	3(100%)	0
	III	44(49%)	1(2%)	0	43(98%)
		Kappa=0.355, P=0.0001			

*ACCF/AHA: American College of Cardiology Foundation/American Heart Association; ESC: European Society of Cardiology.*

**Figure 1:** – *Discrepancy between the 2011 ACCF/AHA and the 2014 ESC guidelines on sudden cardiac death primary prevention in hypertrophic cardiomyopathy* ICD = implantable cardioverter defibrillator, SCD = sudden cardiac death, HCM = hypertrophic cardiomyopathy, ACCF= American College of Cardiology Foundation, AHA = American Heart Association, ESC = European Society of Cardiology, NSVT = non-sustained ventricular tachycardia, BP = blood pressure;^1^Modifier factors: 1. Left ventricular outflow tract gradient ≥30 mmHg; 2. Late gadolineum enhancement on cardiac magnetic resonance; 3.Left ventricular apical aneurysm; 4. Malignant genetic mutation.

The mean calculated ESC risk score was 3±1.7% in the 8 (9%) patients experiencing SCD or appropriate shock. Four (50%) had class IIa recommendation for device implantation with the 2011 ACCF/AHA guideline, but none achieved this class of recommendation with the 2014 ESC model, although 2 (25%) remained in class IIb.

The combination of risk factors that received class IIa recommendation with the 2011 ACCF/AHA strategy was associated with a downgrade in ICD recommendation with the 2014 ESC model (p=0.05). Family history of SCD and NSVT associated with LVOT obstruction were the predictors that showed the greatest decrease of ICD class recommendation under the European guideline ( Table 6 ).

**Table 6:** – Sudden cardiac death risk profile in patients with hypertrophic cardiomyopathy in class IIa for implantable cardioverter defibrillator with the 2011 American College of Cardiology Foundation/American Heart Association guideline: restratification with the 2014 European Society of Cardiology model

	2011 ACCF/AHA / 2014 ESC		

	IIa2011/IIa2014	IIa2011/IIb2014	IIa2011/III2014
	n = 11 (26%)	n = 8 (19%)	n = 24 (55%)
Isolated family history of SCD	2 (12%)	2 (12%)	13 (76%)
Isolated syncope	5 (45%)	1 (10%)	5 (45%)
Syncope + family history of SCD	2 (40%)	2 (40%)	1 (20%)
Family history of SCD + MLVWT ≥30mm	1 (100%)	0	0
NSVT + LVOT obstruction	1 (17%)	3 (50%)	2 (33%)
Abnormal BP response + LVOT obstruction + LGE on CMR	0	0	3 (100%)
	P = 0.05		

*ACCF: American College of Cardiology Foundation; AHA: American Heart Association; ESC: European Society of Cardiology; SCD: sudden cardiac death; MLVWT: maximal left ventricular wall thickness; NSVT: non-sustained ventricular tachycardia; LVOT: left ventricular outflow tract; BP: blood pressure; LGE: late gadolinium enhancement; CMR: cardiac magnetic resonance.*

## Discussion

In this study, for the first time, we compared the SCD primary prevention criteria established by the 2011 ACCF/AHA and the 2014 ESC guidelines in a Brazilian HCM population based on a non-referred outpatient clinical cohort. Our results demonstrate low agreement between the two systems regarding the recommendations for primary prevention ICD. The ESC/HCM Risk-SCD score has lowered the class of implant recommendation over ACCF/AHA in 36% of the patients. Among those in class IIa in the North American guideline, the ESC risk score decreased the class of device recommendation in 74% of the patients, determined an ICD unwarranted in 56% and maintained the recommendation in only 26%. The European risk score has added recommendation in only 1% of the patients. In almost all cases, in which the implantation was not recommended with the North American guideline, the European criteria reassured the decision. The new model has excluded, from class IIa, the 8 (9%) patients experiencing SCD or ICD appropriate shock along the observation period, although 25% of them remained in class IIb.

HCM is an arrhythmogenic heart disease, whose histopathological substrate characterized by hypertrophy, cell disarray, fibrosis and coronary microvascular disease favors the occurrence of lethal ventricular arrhythmias.^5 , 6 , 19 , 20^ Risk stratification for SCD is based on observational data obtained in very selected populations. It is considered complex, due to the heterogeneous character of the disease, and imperfect because many deaths occur in the absence of risk predictors.^5 - 7 , 21^ The limitations offered by the 2003 and 2011 algorithms have been demonstrated in an international registry showing no difference in appropriate shock rates between patients with one, two, three or more predictors.^22^ A posterior validation analysis of these criteria reports that the incidence of SCD and appropriate discharge do not differ between patients with none or only one predictor and that the initial algorithms have limited power to discriminate between high and low risk and could result in unnecessary implants.^23^

This study evaluated a HCM cohort with more advanced age and low risk profile: 78% of the patients remained in functional class I/II, 47% presented no risk factors and 35% showed only one. HCM patients aged ≥60 demonstrate reduced morbidity-mortality and SCD rates, even in the presence of risk predictors.^6^ Five and ten-year SCD and ICD appropriate shock-free survival rates achieved 93% and 92%, respectively, and only 9% of the patients experienced these events along the period. A multicenter longitudinal study presents similar results and supports that HCM, when conveniently treated, shows reduced mortality in adulthood with a ten-year survival rate similar to that expected in the general population.^3^

The mean calculated ESC HCM risk-SCD score of 3.2±2.5 characterized 75% of the patients as low risk. NSVT, syncope and increased MLVWT were more frequent in high-risk patients compared to others.

In this study, we determine the percentages achieved by each one of the score predictors in the tree risk categories with the purpose of discriminating those that reached more weight in order to justify the low agreement between the two guidelines. We ascertained that the factors that have mainly contributed to the calculation and reached increased values in the low, intermediate and high-risk levels were MLVWT, left atrial diameter and age, the latter with a subtractive effect. These findings may justify the low agreement between the two guidelines, considering that MLVWT as a continuous variable, left atrial diameter and age are not included in the North American strategy. Family history of SCD or syncope, both considered as an ACCF/AHA recommendation for ICD therapy, showed lower contribution to the score calculation.

The combination of risk factors characterized as ACCF/AHA class IIa recommendation for ICD in the sample, mainly family history of SCD and NSVT added to LVOT obstruction, was associated to a decrease of device recommendations with the ESC risk score, reaching an ICD unwarranted in 55% of the cases. Our results suggest that the downgrade in ICD recommendations provided by the ESC model is mainly related to cases in which the recommendation with the North American guideline is based on the presence of a single predictor associated or not with a modifying risk factor. These findings are justified by the fact that the European model defines primary prevention on the basis of a set of risk factors and not on the presence of a single marker.

The ESC HCM risk-SCD score has been independently validated in the populations of three continents in observational studies mostly showing that the new model contributes to the improvement of risk stratification and clinical decision-making.^24 - 28^ Other studies point out the sensitivity of low score for the recognition of high-risk patients, the capacity to identify cases with an ICD unwarranted and the similar event rates observed in the three risk levels.^29 - 32^ Our study supports these findings demonstrating that the European model decreases the ICD recommendations compared to the North American guideline, leaves all patients with SCD or appropriate shock unprotected and establishes major agreement in cases not requiring implantation. Nevertheless, the metanalysis of six studies conducted with 7,291 patients demonstrates that in most cases, the five-year SCD risk is properly estimated with the ESC score.^33^

The European score settles the stratification for SCD using a rigid statistical model in a complex disease with unpredictable course. Methodological limitations may depend on left atrial evaluation with diameter, LVOT obstruction with Valsalva maneuver, and on exclusion of myocardial ischemia, late gadolinium enhancement and LV apical aneurysm. Although restrictions may be admitted to its performance, particularly in high-risk patients, the European score should be assimilated in clinical practice as a validated tool to guide therapeutic decisions. The assessment of the percentages achieved by the variables in the formula in each case may contribute to the interpretation of results in clinical practice. In the present study, the North American approach would protect a higher number of individuals than the European criteria, although it could result in unnecessary implants and could expose these populations to device complications such as infections and inappropriate shocks.^4 , 15 , 17^ Prospective studies equally validated in lower risk populations are necessary to identify new predisposing factors that may improve the indications of primary prevention ICD.

### Study limitations

The present study is based on the evaluation of a well-documented single-center HCM cohort comprising a less selected and older population. The clinical characteristics and the reduced event rates show the low-risk profile of the study patients, who differ from those included in the majority of the validation cohorts. These aspects may limit our conclusions to populations presenting the same characteristics. However, the study cases are as representative of the disease as those selected in referral centers with higher risk and more prone to complications.

## Conclusions

In the study of an older low-risk HCM cohort, we found low agreement between the SCD primary prevention criteria established by the 2011 ACCF/AHA and the 2014 ESC guidelines. The ESC HCM risk-SCD score has decreased the ICD recommendation in the study population, especially in those with class IIa under the North American system and left all patients presenting SCD or appropriate shock in the period unprotected. The major contribution for the score calculation of the risk predictors not included in the 2011 ACCF/AHA strategy may justify in some way the discrepancy between the two guidelines.
